# Serum and Urinary Progranulin in Diabetic Kidney Disease

**DOI:** 10.1371/journal.pone.0165177

**Published:** 2016-10-24

**Authors:** Bruna Bellincanta Nicoletto, Thaiana Cirino Krolikowski, Daisy Crispim, Luis Henrique Canani

**Affiliations:** 1 Post Graduate Medical Sciences Program: Endocrinology, School of Medicine, Universidade Federal do Rio Grande do Sul (UFRGS), Porto Alegre, Brazil; 2 Nutrition Course, School of Medicine, Universidade Federal do Rio Grande do Sul (UFRGS), Porto Alegre, Brazil; 3 Division of Endocrinology, Hospital de Clínicas de Porto Alegre, Porto Alegre, Brazil; Hospital Universitario de la Princesa, SPAIN

## Abstract

Progranulin has been recognized as an adipokine related to obesity, insulin resistance and type 2 diabetes mellitus (T2DM). There are scarce data regarding progranulin and kidney disease, but there are some data linking diabetic kidney disease (DKD) and increased progranulin levels. We aimed to better describe the relationship between serum and urinary progranulin levels and DKD in T2DM. This is a case-control study including four groups of subjects: 1) Advanced DKD cases: T2DM patients with estimated glomerular filtration rate (eGFR) <60 mL/min/1.73m^2^; 2) Albuminuric DKD cases: T2DM patients with urinary albumin excretion (UAE) ≥30 mg/g creatinine and eGFR ≥60 mL/min/1.73m^2^; 3) Diabetic controls: T2DM patients with UAE <30 mg/g creatinine and eGFR ≥60 mL/min/1.73m^2^; and 4) Non-diabetic controls: individuals without T2DM. Progranulin was determined by enzyme-linked immunosorbent assay. One hundred and fourteen patients were included (23 advanced DKD cases, 25 albuminuric DKD cases, 40 diabetic controls and 26 non-diabetic controls). Serum progranulin was increased in advanced DKD compared to other groups [70.84 (59.04–83.16) vs. albuminuric cases 57.16 (42.24–67.38), diabetic controls 57.28 (42.08–70.47) and non-diabetic controls 44.54 (41.44–53.32) ng/mL; p<0.001]. Urinary progranulin was decreased in advanced DKD cases compared to albuminuric cases [10.62 (6.30–16.08) vs. 20.94 (12.35–30.22); diabetic controls 14.06 (9.88–20.82) and non-diabetic controls 13.51 (7.94–24.36) ng/mL; p = 0.017]. There was a positive correlation between serum progranulin and body mass index (r = 0.27; p = 0.004), waist circumference (r = 0.25; p = 0.007); body fat percentage (r = 0.20; p = 0.042), high-sensitive C reactive protein (r = 0.35; p<0.001) and interleukin-6 (r = 0.37; p<0.001) and a negative correlation with eGFR (r = -0.22; p = 0.023). Urinary progranulin was positively associated with albuminuria (r = 0.25; p = 0.010). In conclusion, progranulin is affected by a decrease in eGFR, being at a higher concentration in serum and lower in urine of DKD patients with T2DM and eGFR <60 mL/min/1.73m^2^. It is also associated with markers of obesity and inflammation.

## Introduction

Progranulin (PGRN) is a 68–88 kDa protein, also known as acrogranin, proepithelin, granulin-epithelin precursor or PC-cell derived growth factor [1, 2]. It is expressed in many cell types, including immune cells, epithelial cells, neurons, and adipocytes [3]. It was first identified for its growth factor like properties, being involved in early embryogenesis and tissue remodeling, acting as an anti-inflammatory molecule [4, 5]. In the central nervous system, PGRN performs neurotrophic and neuroprotective actions [6]. There is also evidence of PGRN effects on cancer, contributing to tumor proliferation, invasion and cell survival [7–9].

Recently, PGRN was recognized as an adipokine related to obesity and insulin resistance, revealing its metabolic function and pro-inflammatory properties [3, 10]. Elevated serum levels of PGRN are found in obese patients, with a positive correlation with body mass index (BMI) [10, 11]. Moreover, PGRN is associated with body fat, mainly with abdominal depot [11, 12]. Visceral adiposity, in turn, is strongly related to chronic inflammation, due to its secretion of adipokines and immune cell-derived cytokines [13].

PGRN seems to promote interleukin-6 (IL-6) expression, leading to insulin resistance [14]. In humans, there is a positive correlation between serum PGRN and IL-6 and HOMA-IR index (Homeostasis Model Assessment for insulin resistance) [10, 15]. Increased PGRN serum levels have also been described in patients with type 2 diabetes mellitus (T2DM) [10–12].

Diabetic kidney disease (DKD) is a common complication of diabetes [16], associated with cardiovascular disease [17]. Recently, increased serum PGRN was observed in macroalbuminuric patients with T2DM [18]. The theoretical role of PGRN in DKD could be found in a recent review [19]. Moreover, PGRN was described as a renal function-dependent adipokine, since elevated serum levels were observed in patients at stage 5 of chronic kidney disease (CKD) [20]. Urinary levels of PGRN in patients with T2DM and DKD remain unknown. However, in patients with type 1 diabetes, PGRN concentration in urine was predictive of early renal function decline and albuminuria [21].

Therefore, the main objective of this study was to investigate the association of serum and urinary PGRN levels with DKD in T2DM. We also aimed to evaluate the factors associated with PGRN levels.

## Materials and Methods

### Design and patients

One hundred and fourteen patients were included in this case control study. Patients with T2DM and no exclusion criteria were invited to participate in the study. Two study groups were included: 1) Advanced DKD cases: T2DM patients with estimated glomerular filtration rate (eGFR) <60 mL/min/1.73m^2^ and 2) Albuminuric DKD cases: T2DM patients with urinary albumin excretion (UAE) ≥30 mg/g creatinine and eGFR ≥60 mL/min/1.73m^2^. Once cases were included, controls were sought based on similar age, gender and BMI and were divided into another two control groups: 1) Diabetic controls: patients with T2DM with UAE <30 mg/g creatinine and eGFR ≥60 mL/min/1.73m^2^ and 2) Non-diabetic controls: individuals without diabetes and eGFR ≥60 mL/min/1.73m^2^. All groups consisted of outpatients attending the Hospital de Clínicas de Porto Alegre (Rio Grande do Sul, Brazil) between October 2013 and November 2014. Exclusion criteria were age below 18 years old, cancer, pancreatitis, acute infections, secondary T2DM, dialysis, transplantation, pregnancy and alcohol or drug abuse. Fourteen patients refused to participate in the study.

The diagnosis of T2DM and increased UAE was based on American Diabetes Association criteria [22, 23]. Two of three spot urine samples were considered for classification of increased UAE. The eGFR was assessed by the Chronic Kidney Disease Epidemiology Collaboration (CKD-EPI) equation [24].

This study was approved by the Ethics Committee of Hospital de Clínicas de Porto Alegre and all subjects received adequate information about the study and gave their written informed consent.

### Clinical, anthropometric and biochemistry assessment

Demographic and clinical data were collected using a standard questionnaire and review of medical registry, including the following variables: age, gender, T2DM duration, arterial hypertension, systolic and diastolic blood pressure and the use of antidiabetic, antihypertensive and antilipidemic medications. Hypertension was defined by blood pressure ≥140/90 mmHg or antihypertensive medication use.

All anthropometric evaluations were performed by the same dietitian and consisted of weight, height, and waist circumference. Body weight (kg) and height (m) were assessed in order to calculate BMI (kg/m^2^). Waist circumference was measured at the midpoint between the lowest rib and the iliac crest, using a flexible, inelastic measuring tape. Body composition was measured with a direct segmental multiple-frequency bioelectrical impedance analysis method (InBody 230; Biospace, Seoul, Korea) to assess body fat percentage (BF%) and trunk fat (kg). The measurements were performed with the patient fasting, without shoes, wearing light clothing, in a stable condition [25].

Blood and spot urine samples were taken after 12-hour overnight fasting. High-sensitivity C reactive protein (hsCRP), fasting plasma glucose, total cholesterol, HDL-cholesterol, triglycerides, proteinuria, albuminuria and urinary creatinine were determined using standard local laboratory techniques. Serum creatinine was measured by the Jaffe method (Modular P, Roche Diagnostic, Mannheim, Germany) traceable to isotope dilution mass spectrometry [26]. HbA1c was measured by the HPLC method (Bio-Rad Variant™ II Turbo analyzer), as standardized by the National Glycohemoglobin Standardization Program (http://www.ngsp.org/certified.asp) and aligned with the International Federation of Clinical Chemistry [27]. The Clinical Pathology Department participates in an HbA1c External Quality Assurance Program with excellent performance. LDL-cholesterol was calculated using the Friedewald formula when triglyceride levels were lower than 400 mg/dL.

Blood and urine were centrifuged, and samples obtained were stored in duplicates at -80°C for later PGRN and IL-6 analysis. The PGRN concentration was determined in serum and urine samples, using the Human Progranulin Quantikine ELISA kit (R&D Systems, Minneapolis, MN, USA). Of all samples (serum and urine), 30.7% were performed in duplicates. The assay sensitivity was 0.54 ng/mL and assay range was 1.56–100 ng/mL, whereas the inter-assay coefficient was less than 10% for serum and urine samples. The IL-6 concentration was assessed in serum samples by Human IL-6 Quantikine ELISA kit (R&D Systems, Minneapolis, MN, USA). Duplicates were performed in 34.2% of serum samples. The assay sensitivity was 0.7 pg/mL, with a range between 3.12–300 pg/mL and inter-assay coefficient less than 6.5%.

### Statistical analyses

The sample size calculation was based on previous studies [10, 12, 20], in which we observed an approximate difference of one standard deviation in PGRN serum levels between groups of interest, and a correlation of 0.3 between serum PGRN and some biochemical markers. Therefore, considering α = 0.05 and β = 0.10 errors, the total sample estimated was 113 individuals.

Data were analyzed using the Statistical Package for Social Sciences version 20.0 program (SPSS, Chicago, IL). After assessing normality of continuous variables by the Shapiro Wilk test, the study groups were compared by One-Way Analysis of Variances (ANOVA) with Levene and Tukey or Kruskal-Wallis with Dunn tests, as appropriate. Data with normal distribution are presented as mean ± SD, whereas data with asymmetric distribution are presented as median (interquartile range). Categorical variables were compared among groups by Chi-square test and they are reported as absolute numbers and percentages. Correlations were tested by Spearman’s correlation coefficient, since the serum and urinary PGRN variables presented an asymmetric distribution. Multivariate linear regression analyses were performed, using serum or urinary PGRN as dependent variables. The independent variables included in each model were selected if they were correlated with PGRN using Spearman’s correlation coefficient and had no collinearity. Normal distribution of residuals was accepted in multivariate linear regression analyses. Only valid cases were included in each analysis. The level of statistical significance was established as 5%.

## Results

### Clinical characteristics

Patients’ demographic and clinical characteristics are shown in Table 1. Insulin was the main hypoglycemic agent used by advanced cases patients, while other T2DM groups were treated mainly with oral agents, such as metformin and glibenclamide. Patients with diabetes also presented more hypertension and use of anti-hypertensive medication and statins than the non-diabetic group. BMI and body composition assessed by BF% and trunk fat were similar in the four study groups. Waist circumference was lower in non-diabetic subjects when compared to advanced DKD patients. Non-diabetic subjects had higher LDL-cholesterol, while advanced DKD cases had worse HDL-cholesterol and triglyceride levels. Elevated IL-6 was also observed in the advanced DKD group (Table 1).

**Table 1:** Clinical and laboratory characteristics of study subjects.

	Non-diabetic controls (n = 26)	Diabetic controls (n = 40)	Albuminuric DKD cases (n = 25)	Advanced DKD cases (n = 23)	P value
Age (years)	58.8 ± 10.8	59.8 ± 8.2	63.3 ± 7.9	61.5 ± 9.8	0.290
Male gender, n (%)	12 (46.2)	19 (47.5)	11 (44.0)	12 (52.2)	0.952
Diabetes mellitus duration (years)	-	14.9 ± 9.9	14.2 ± 7.8	18.0 ± 9.1	0.307
Antidiabetic agents, n (%)					
Insulin	-	22 (55.0) ^a^	18 (72.0) ^ab^	23 (100) ^b^	0.001
Metformin	-	34 (85.0) ^a^	25 (100) ^a^	4 (17.4) ^b^	<0.001
Glibenclamide	-	16 (40.0) ^a^	10 (40.0) ^a^	2 (8.7) ^b^	<0.001
Statin use, n (%)	4 (15.4) ^a^	27 (67.5) ^b^	21 (84.0) ^b^	22 (95.7) ^b^	<0.001
Anti-hypertensive medication use, n (%)	10 (38.5) ^a^	37 (92.5) ^b^	25 (100) ^b^	23 (100) ^b^	<0.001
Hypertension n (%)	12 (52.2) ^a^	38 (95.0) ^b^	25 (100) ^b^	23 (100) ^b^	<0.001
Systolic blood pressure (mmHg)	130.8 ± 14.7	137.2 ± 21.4	139.5 ± 14.2	144.7 ± 20.6	0.087
Diastolic blood pressure (mmHg)	78.6 ± 10.1	80.5 ± 12.3	79.5 ± 11.9	82.9 ± 13.2	0.645
Body mass index (kg/m^2^)	28.7 (25.5–32.0)	30.8 (26.8–35.8)	31.8 (27.4–36.7)	30.9 (28.0–38.5)	0.212
Waist circumference (cm)	99.8 ± 13.3 ^a^	105.1 ± 13.7 ^ab^	109.2 ± 12.0 ^ab^	111.0 ± 18.9 ^b^	0.036
Body fat %	36.50 ± 9.44	36.47 ± 9.20	36.53 ± 11.11	37.66 ± 11.84	0.976
Trunk fat (kg)	15.42 ± 5.82	16.27 ± 5.42	16.20 ± 5.16	16.19 ± 6.58	0.954
Fasting plasma glucose (mg/dL)	90.0 (84.3–94.0) ^a^	141.5 (114.8–170.5) ^b^	166 (94.5–227.5) ^b^	139 (97–178) ^b^	<0.001
HbA1c (%)	5.6 (5.3–5.7) ^a^	7.9 (6.9–9.2) ^b^	8.7 (7.6–9.4) ^b^	7.9 (7.2–9.4) ^b^	<0.001
HbA1c (mmol/mol)	38 (34–39) ^a^	63 (52–77) ^b^	72 (60–79) ^b^	63 (55–79) ^b^	<0.001
Total cholesterol (mg/dL)	188 (165.8–215.8)	172.5 (145.3–193.8)	172 (151–200.5)	170 (147–213)	0.102
LDL-cholesterol (mg/dL)	122.4 (101.1–142.9) ^a^	102.1 (79–124.4) ^ab^	92.4 (79.4–99) ^bc^	89 (72–122.5) ^bc^	0.002
HDL-cholesterol (mg/dL)	46.0 (38.8–51.3) ^a^	40.5 (35.0–45.8) ^ab^	37.0 (30.0–44.0) ^b^	36.0 (30.0–44.0) ^b^	0.004
Triglycerides (mg/dL)	127.5 (84.3–168.5) ^a^	139.0 (96.0–192.8) ^ab^	167.0 (122.5–298.5) ^bc^	223.0 (148.0–288.0) ^c^	0.001
hsCRP (mg/dL)	3.34 (1.81–10.80)	3.44 (1.13–8.06)	2.71 (1.78–7.04)	6.06 (1.89–18.56)	0.382
IL-6 (pg/mL)	3.12 (3.12–3.17) ^a^	3.12 (3.12–3.94) ^a^	3.12 (3.12–4.06) ^a^	7.35 (4.18–10.27) ^b^	<0.001
eGFR (mL/min/1.73m^2^)	97.2 (78.7–109.8) ^a^	95.6 (86.1–115.8) ^a^	98.0 (88.0–104.5) ^a^	23.0 (17.0–33.6) ^b^	<0.001
Albuminuria (mg/L)	7.4 (3.0–12.3) ^a^	10.7 (4.58–18.93) ^a^	100.5 (63.55–181.5) ^b^	459.2 (186.2–1561) ^b^	<0.001
UAE (mg albumin/g creatinine)	5.87 (3.78–8.46) ^a^	7.32 (4.24–16.12) ^a^	81.92 (43.02–168.13) ^b^	718.7 (157.8–2142) ^b^	<0.001
Proteinuria (mg/L)	80 (40–180) ^a^	60 (70–108) ^a^	250 (180–350) ^b^	880 (400–2290) ^b^	<0.001

hsCRP: high-sensitivity C reactive protein; IL-6: interleukin-6; eGFR: estimated glomerular filtration rate; UAE: urinary albumin excretion.

### Serum and urinary PGRN concentrations

**Fig 1:**
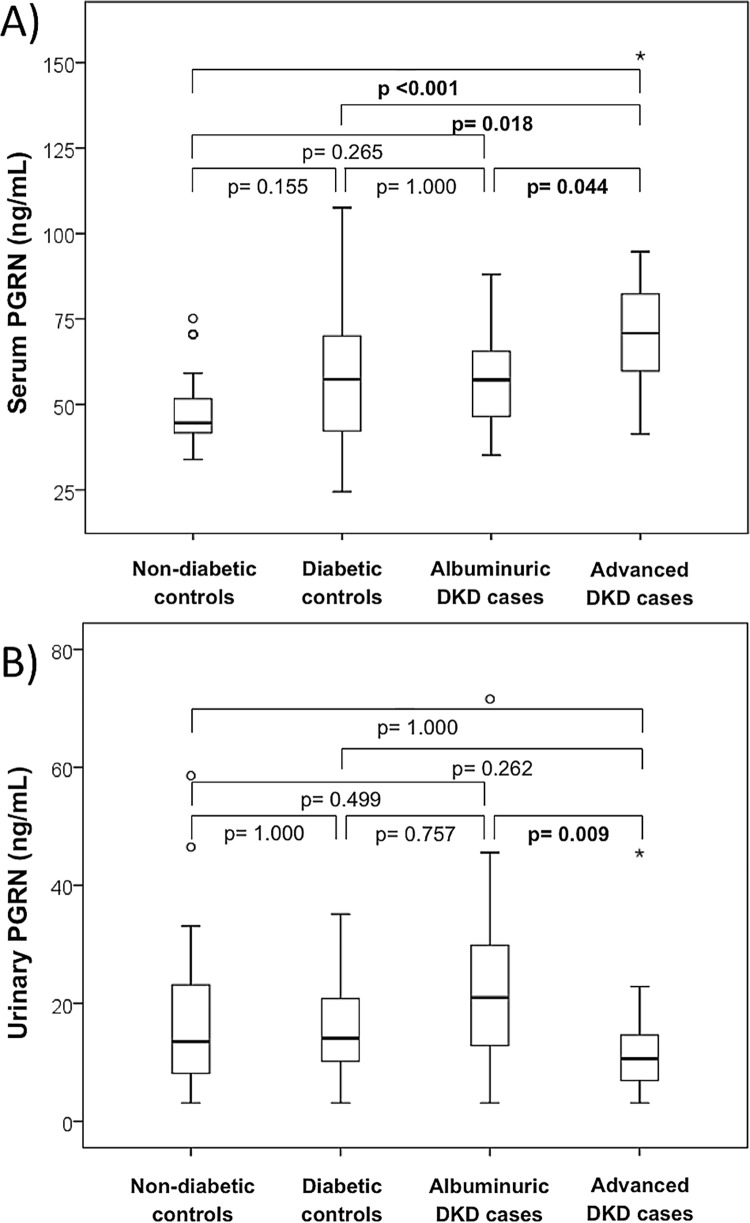
**Boxplots showing serum (A) and urinary (B) levels of PGRN (ng/mL) according to study groups.** PGRN: progranulin; DKD: diabetic kidney disease.

Serum PGRN was increased in advanced DKD patients compared to the other groups [(70.84 (59.04–83.16) vs. albuminuric DKD cases 57.16 (42.24–67.38), diabetic controls 57.28 (42.08–70.47) and non-diabetic controls 44.54 (41.44–53.32) ng/mL; p<0.001] (Fig 1A). There was no difference in PGRN serum levels between albuminuric DKD cases, diabetic controls and non-diabetic groups. However, the nominal values for the diabetic groups seemed to be higher than for the non-diabetic group. To evaluate whether this was due to sample size effect, we analyzed diabetic controls and albuminuric DKD patients together versus subjects without diabetes. PGRN serum levels in the first group [57.16 (42.62–69.18) ng/mL; n = 65] were significantly higher than non-diabetic subjects [44.54 (41.44–53.32) ng/mL; n = 26; p = 0.014].

Urinary PGRN was decreased in advanced DKD cases when compared to albuminuric DKD patients [10.62 (6.30–16.08) vs. 20.94 (12.35–30.22); diabetic controls 14.06 (9.88–20.82) and non-diabetic controls 13.51 (7.94–24.36) ng/mL; p = 0.017] (Fig 1B). The other groups presented similar levels of PGRN in urine. Urinary PGRN levels were not available for seven patients, so this analysis was performed including 107 individuals.

### Correlations

The relationships of serum and urine PGRN were evaluated using Spearman’s correlation test (Table 2). Taking the entire group, the serum PGRN correlated with BMI (r = 0.27; p = 0.004), waist circumference (r = 0.25; p = 0.007), BF% (r = 0.20; p = 0.042); hsCRP (r = 0.35; p <0.001), IL-6 (r = 0.37; p<0.001), albuminuria (r = 0.25; p = 0.008) and proteinuria (r = 0.24; p = 0.010). A negative correlation with eGFR (r = -0.22; p = 0.023) was observed (Table 2). In multivariate linear regression, serum PGRN was inversely and independently associated with eGFR (beta = -0.28; p = 0.006) (Table 3). The interaction between diabetes and eGFR was analyzed in the multivariate model, and no association was observed (beta = 0.25, p = 0.761).

**Table 2:** Correlations between serum / urinary PGRN and other parameters.

All patients	Serum PGRN r (P) (n = 114)	Urinary PGRN r (P) (n = 107)
Diabetes mellitus duration (years)	0.06 (0.555)	-0.07 (0.551)
Systolic blood pressure (mmHg)	0.01 (0.900)	-0.02 (0.863)
Diastolic blood pressure (mmHg)	0.04 (0.655)	0.17 (0.096)
Body mass index (kg/m^2^)	0.27 (0.004)	0.12 (0.219)
Waist circumference (cm)	0.25 (0.007)	0.16 (0.091)
Body fat %	0.20 (0.042)	-0.01 (0.901)
Trunk fat (kg)	0.16 (0.117)	0.10 (0.300)
Fasting plasma glucose (mg/dL)	0.13 (0.158)	0.06 (0.551)
HbA1c (%, mmol/mol)	0.16 (0.095)	0.05 (0.611)
Total cholesterol (mg/dL)	0.03 (0.743)	0.01 (0.966)
LDL-cholesterol (mg/dL)	-0.08 (0.424)	-0.01 (0.951)
HDL-cholesterol (mg/dL)	-0.05 (0.622)	-0.14 (0.165)
Triglycerides (mg/dL)	0.15 (0.120)	0.08 (0.407)
hsCRP (mg/dL)	0.35 (<0.001)	0.18 (0.071)
IL-6 (pg/mL)	0.37 (<0.001)	-0.06 (0.553)
eGFR (mL/min/1.73m^2^)	-0.22 (0.023)	0.16 (0.101)
Albuminuria (mg/L)	0.25 (0.008)	0.25 (0.010)
Proteinuria (mg/L)	0.24 (0.010)	0.38 (<0.001)

PGRN: progranulin; hsCRP: high-sensitivity C reactive protein; IL-6: interleukin-6; eGFR: estimated glomerular filtration rate.

**Table 3:** Multivariate linear regression analysis models.

Variable		Beta	P value
**Dependent variable: Serum PGRN**			
	**All sample (n = 106)**		
	Age (years)	-0.14	0.139
	Male gender	0.12	0.060
	Body mass index (kg/m^2^)	0.05	0.595
	hsCRP (mg/dL)	0.12	0.244
	IL-6 (pg/mL)	0.09	0.364
	eGFR (mL/min/1.73m^2^)	-0.28	0.006
	Type 2 diabetes mellitus	0.16	0.095
	**Individuals with eGFR ≥ 60 mL/min/1.73m**^**2**^ **(n = 90)**		
	Age (years)	-0.07	0.462
	Male gender	0.08	0.422
	Body mass index (kg/m^2^)	0.30	0.004
	hsCRP (mg/dL)	0.15	0.139
	Type 2 diabetes mellitus	0.21	0.040
**Dependent variable: Urinary PGRN**			
	**All sample (n = 107)**		
	Age (years)	-0.02	0.813
	Male gender	-0.07	0.465
	Albuminuria (mg/L)	0.28	0.013
	eGFR (mL/min/1.73m^2^)	0.34	0.004
	Type 2 diabetes mellitus	0.19	0.848

PGRN: progranulin; hsCRP: high-sensitivity C reactive protein; IL-6: interleukin-6; eGFR: estimated glomerular filtration rate.

To identify associations between serum PGRN and other covariates in patients with preserved renal filtration, we performed Spearman’s correlation test excluding the advanced DKD group. Serum PGRN remained associated with BMI (r = 0.32; p = 0.002), waist circumference (r = 0.28; p = 0.007), BF% (r = 0.29; p = 0.008) and hsCRP (r = 0.35; p <0.001), and was also associated with trunk fat (r = 0.27; p = 0.016). In a multivariate linear regression excluding the advanced DKD group, serum PGRN remained associated with BMI (beta = 0.30; p = 0.004), and was also associated with T2DM (beta = 0.21; p = 0.040), independently of age, gender and hsCRP (Table 3).

Urinary PGRN was positively associated with albuminuria (r = 0.25; p = 0.010) and proteinuria (r = 0.38; p <0.001) (Table 2). In multivariate linear regression analysis, albuminuria was associated with urinary PGRN (beta = 0.28; p = 0.013) independently of eGFR, age, gender and T2DM (Table 3). Serum and urinary PGRN correlations with UAE were maintained. There was no correlation between serum and urinary PGRN (r = 0.08; p = 0.400).

## Discussion

In the present study, we were able to better characterize serum and urinary PGRN levels in patients with T2DM according to renal function. Patients with diabetes and eGFR <60 mL/min/1.73m^2^ presented elevated serum and reduced urinary levels of PGRN. Moreover, this adipokine was higher in serum of T2DM patients when compared to non-diabetic subjects, independently of renal function. Correlations with inflammatory, adiposity and renal function markers were also described.

Previous data regarding PGRN and kidney disease are reported by few studies. Xu et al. [18] found elevated serum PGRN concentrations in patients with T2DM and macroalbuminuria (UAE rate >300 mg/24h). However, that subset of patients also presented reduced eGFR [18]. In a recent study evaluating 532 patients with CKD stages 1–5, Richter et al. [20] observed that PGRN serum levels significantly increased with deterioration of renal function. Besides, in our sample, the elevated serum PGRN concentration among subjects with advanced DKD was accompanied with a low PGRN in urine. Despite we did not find a correlation between serum and urinary levels of PGRN, in agreement with Richter et al., who also did not find it in a subgroup of their study population [20, 28], it is supposed that renal filtration is an important route of PGRN elimination [28].

There is evidence that PGRN expression in the kidney is reduced in mice models of acute kidney injury [29] and DKD [30]; however, circulating PGRN is increased [29, 30]. In humans, higher levels of serum PGRN are observed in end-stage CKD [20] and also after nephrectomy, a model of acute kidney insufficiency [28]. Moreover, a negative correlation between serum PGRN and eGFR has been previously reported in the literature [18, 20], even as we observed in the present study. Therefore, the relationship of PGRN with kidney disease appears to be its accumulation due to the eGFR decrease, suggesting renal clearance as a route of PGRN elimination. It corroborates our findings of higher PGRN in serum and lower in urine of advanced DKD cases.

On the other hand, increased circulating PGRN might be a compensatory mechanism to reduce renal deterioration, since it was demonstrated that PGRN could attenuate inflammation in an acute condition [29]. In a mouse model of renal ischemia-reperfusion injury, Zhou et al. [29] observed that PGRN deficiency was associated with higher elevation of serum creatinine and blood urea nitrogen, more severe morphological injury and higher inflammatory response. The administration of recombinant PGRN in vitro attenuated inflammation, exerting a protective role in acute kidney injury [29]. However, it is speculated that PGRN could play different functions in different metabolic conditions [19] and further studies are needed to understand whether increased serum PGRN in advanced DKD in T2DM patients could play an anti-inflammatory role or whether its accumulation in serum is only due to the decreased eGFR.

To our knowledge, this is the first study evaluating the association of urinary PGRN and DKD in T2DM. Previously, Schlatzer et al. [21] investigated PGRN in urine of 74 patients with type 1 diabetes mellitus and concluded that a panel of three proteins (Tamms-Horsfall glycoprotein, clusterin and human α-1 acid glycoprotein) plus PGRN could be used to predict early signs of DKD [21]. The present study design does not allow us to identify urinary PGRN as an early marker of DKD. However, our findings show a positive correlation of urinary PGRN with albuminuria. Albumin is a protein of 67 kDa [31], a very similar size to PGRN (66–88 kDa) [1]. Possibly, their similar molecular size affects their renal clearance, but when eGFR decreases at <60 mL/min/1.73m^2^, PGRN may accumulate in serum (advanced DKD patients presented higher PGRN in serum and lower in urine). However, the renal mechanisms possibly involved are still unknown.

Studies regarding PGRN in diabetes should consider the eGFR, since kidney disease is a common complication that could influence the result. Previous data comparing patients with T2DM to non-diabetic subjects have reported elevated serum PGRN associated with the disease [10–12]. The studies of Tönjes et al. [12] and Youn et al. [11] observed increased serum PGRN in patients with T2DM; however they did not consider eGFR in their analysis. On the other hand, Qu et al. [10] excluded patients with renal disease from their study and, even so, observed elevated serum PGRN in patients with T2DM, independently of obesity. Lastly, Xu et al. [18] did not find a significant difference in serum PGRN when comparing non-diabetic subjects with normoalbuminuric T2DM patients. In the present study, serum PGRN differences among albuminuric DKD cases, diabetic controls and non-diabetic groups did not reach statistical significance. But when diabetic controls and albuminuric DKD patients were grouped and compared to subjects without diabetes, we observed that serum PGRN are higher in patients with diabetes, independently of kidney disease.

The association of PGRN in diabetes is in accordance with its putative role in insulin resistance [14, 19]. Experimental studies reported that PGRN promotes IL-6 expression, impacting on insulin signaling [14]. The adipokine PGRN has been previously associated with inflammatory markers, as hsCRP [11, 15] and IL-6 [10, 15, 18]. In our study, we also observed a positive correlation between serum PGRN, hsCRP and IL-6, corroborating the pro-inflammatory effects previously suggested for PGRN [3, 10]. There is also some evidence supporting a correlation of PGRN with HbA1C and fasting plasma glucose [10, 11, 32]; however, these associations were not observed by Xu et al. [18], even as we did not observe them in the present study.

PGRN is secreted by adipocytes [3] and acts as a chemoattractant molecule which bring monocytes into adipose tissue, favoring chronic inflammation, obesity and its consequences [11]. Previous studies report the association of PGRN with obesity, with higher serum levels in obese subjects, independently of diabetes [10, 11]. In our sample, serum PGRN was associated with BMI and also with other measurements of adiposity such as waist circumference and BF%, corroborating previous data [10–12, 32].

This study provides a comprehensive and integrated evaluation of a new adipokine in DKD in T2DM. However, there are some limitations. First, the cross-sectional design does not allow an investigation of a causative role between DKD development and changes in PGRN levels. Second, the sample size is relatively small, but we had power to conduct the study, based on previous sample size calculation. Indeed, secondary analyses must be carefully interpreted. Further studies with a longitudinal design are necessary to investigate the association of urinary PGRN levels and DKD in T2DM.

In conclusion, our results suggest that serum PGRN depends on eGFR. Serum PGRN is elevated among patients with low eGFR and urinary PGRN correlates with albuminuria. Furthermore, PGRN correlates with adiposity and inflammation markers, and is associated with T2DM. Prospective studies are needed to find out whether the PGRN might be related to the prognosis of DKD.
